# Effectiveness and working mechanisms of the InConnection approach in multi-problem families: study protocol of a mixed-methods study

**DOI:** 10.1186/s12913-020-05553-3

**Published:** 2020-07-25

**Authors:** Natasha Koper, Hanneke E. Creemers, Susan Branje, Geert Jan J. M. Stams, Levi van Dam

**Affiliations:** 1 Department of Youth and Family, Utrecht University, PO box 80140, 3508TC Utrecht, the Netherlands; 2 Department of Forensic Child and Youth Care Sciences, University of Amsterdam, Amsterdam, The Netherlands; 3 YIM Foundation, Amersfoort, The Netherlands; 4 Spirit youth care, Amsterdam, The Netherlands

**Keywords:** Effectiveness, InConnection approach, Mixed-method, Multi-problem youth, Quasi-experimental trial, Youth initiated mentoring

## Abstract

**Background:**

Multi-problem families face problems in several domains that are often found to be chronic and intergenerational. Effective mental health care for youth from these families is currently lacking, urging research on new methods. The InConnection approach is an integrated care program to improve resilience in multi-problem families by connecting the professional expertise from multiple disciplines with the informal social network of the youth. Specifically, youth are asked to nominate a *youth initiated mentor* (YIM) from among the supportive adults in their network. The aim of this protocol is to describe the design of a mixed-methods study to examine the effectiveness and working mechanisms of the InConnection approach.

**Method/design:**

The effectiveness of the InConnection approach is studied in a quasi-experimental questionnaire study using propensity score matching, with *N* = 300 families with youth aged 10–23 years receiving treatment in either the intervention group (InConnection approach) or the control group (care as usual). The main outcome variables include youth resilience (*primary*), youth mental health, parental functioning, and the number, duration and types of out-of-home placements. Mediators, moderators, and predictors of effectiveness are examined. Assessments take place at the start of the care program and after three, nine and 15 months. Additionally, semi-structured interviews are conducted with families who have and have not nominated a YIM to understand why some families successfully nominate a YIM, whereas others do not.

**Discussion:**

Effective care for youth in multi-problem families is urgently needed. Given its flexibility and accessibility to suit all youth aged 10–23 years from multi-problem families, and its low costs compared to out-of-home placements, the InConnection approach seems an appealing approach to support these families. The current study will provide information on the effectiveness of the InConnection approach. Strengths of this study include its robust design, the ecological validity, and the inclusion of possible mediators, predictors, and moderators of treatment effects.

**Trial registration:**

Netherlands Trial Register NL7565. Retrospectively registered on March 5, 2019.

## Background

Multi-problem families face several problems, which are often chronic and intergenerational, and which occur in multiple domains, such as psychosocial functioning, family functioning, mental health, financial situation and functioning in their social networks [1, 2]. Such problems may place the child’s development at risk [3]: Children in multi-problem families experience more internalizing and externalizing behavior problems and a lower quality of life compared to children in the general population [1]. Not surprisingly, both parents and children in multi-problem families receive more mental health care, have a longer history of care, and receive more intensive care, such as out-of-home placements, than parents and children in the general population [1]. Despite the frequency and intensity of care offered to multi-problem families, there is no convincing evidence for the effectiveness of care for youth in multi-problem families in general [4], nor for (residential) out-of-home care for youth in particular [5, 6]. Given the severe and chronic difficulties faced by multi-problem families and the lack of effect of existing treatment programs for these families, evidence-based care approaches are urgently needed.

### Care as usual for multi-problem families

Treatment for multi-problem families is commonly systemic or family based. These treatment programs generally provide individualized care in multiple domains, strive to actively involve the family system in decision making, and take place in the least restrictive environment [7]. Given the complexity of problems, multi-problem families often receive support from different care providers. This may result in fragmentation of care, hampered coordination between professionals and institutions, and single solutions for complex problems [8–10]. To avoid this, treatment approaches have been developed in which various forms of care can be integrated and coordinated by a case manager or family guardian who functions as the link between the family and professional care services. Examples are the ‘Wraparound care’ model in the United States [11], the ‘Troubled Families’ program in the United Kingdom [12], and the ‘One family, one plan’ policy in the Netherlands [13]. These approaches and policies integrate *formal* care systems, that is, care provided by organizations in formal settings (e.g., health care and social services), yet very few integrate formal with *informal* care systems, that is, a family’s informal social network including family, friends and informal groups. As multi-problem families attract various support systems, including informal support, and strong social support networks are linked to higher levels of resilience [14], that is, successful adaption in face of adversity [15], treatment programs could be enhanced by promoting the coordination between formal and informal support [10], thus using the full potential of families’ support systems.

### The InConnection approach

An innovative approach has been developed that addresses this potential by actively involving a youth initiated mentor (YIM) from the youth’s social network: the InConnection approach [16, 17]. The InConnection approach is a specialized care approach, and aims to increase resilience in youth in multi-problem families and prevent (repetition of) out-of-home placements. The approach has two features that distinguish the approach from care as usual for multi-problem families [18]. First, it involves care provided by a multidisciplinary team, consisting of professionals specialized in youth and family care, psychiatry, addiction care, and care for people with mild intellectual disabilities. The InConnection approach thereby extends other approaches, as it does not only include a case manager who coordinates care from different organizations or types of expertise, but brings the different types of expertise and care together within one approach and team. This approach thus offers families direct access to a wide range of specialized treatment possibilities, depending on the family’s needs [17]. Examples are youth-focused treatments, such as cognitive behavioral therapy, psychomotor therapy; caregiver and family-focused treatments, such as, parent training, trauma therapy; and multisystem treatments, such as, multisystemic therapy. Despite the different forms of treatment, families experience continuity of care as treatments are coherently organized to meet the family’s needs and preferences [19]. Integrating (mental) health care is considered to improve treatment effect and efficiency, quality of life, and client satisfaction [19].

Second, the InConnection approach includes an innovative method to collaborate with the youth’s social network. In the first phase of the treatment, youth nominate a YIM from the supportive adults within their social networks. The YIM is a confidant and spokesperson for the youth, and a partner for parents and professionals [20]. During treatment all members of the client system, including the YIM, actively participate in the decision-making process by giving their perspectives on desired treatment goals and contributing to reaching these goals [17]. The active participation of the client system stimulated by the InConnection approach is what makes the approach more client-focused and strength-based than care as usual. Moreover, the role of an InConnection case manager is to guide and facilitate a collaborative process that contributes to sustainable improvements, rather than directly addressing the problems in a family. As a result, the contact time of an InConnection case manager is on average 6 h per week [17], as compared to 10–20 h per week in care as usual.

The InConnection approach assumes that all youth have a mentor who they can nominate as YIM. Approximately 83% of multi-problem youth treated with the InConnection approach found a YIM within 33 days [16], suggesting that most youth do indeed have supportive adults in their social networks. Youth nominate a mentor based on aspects like personality, trustworthiness, and similarities in experiences [21], yet is it not known why some youth do not nominate a YIM [16]. It is possible that these youth do not have bonds with adults that meet their criteria for being a YIM, or that youth may not be willing to disclose information about their problems and engagement in treatment to non-parental adults due to a lack of trust. Compared to non-clinical youth, youth of multi-problem families are more likely to have insecure attachment representations [22], and therefore experience less trust in relationships [23]. To our knowledge, there is no research to date on what makes families successful in nominating a YIM.

### Effectiveness of the InConnection approach

The potential of mentoring for enhancement of treatment effectiveness has been empirically supported. Research indicates that the mere presence of a mentor and participation in mentoring programs are positively associated with positive youth outcomes [24, 25], including resilience [26]. For example, youth who participated in treatment programs in which they nominated YIMs demonstrated better academic and vocational outcomes [27], and reduced mortality rates [28] after participation. In addition, preliminary positive results of the InConnection approach, including working with YIMs, have been found. In two studies with a total of 138 youth from multi-problem families, approximately 80–90% of youth continued to receive outpatient treatment only, despite a prior indication for out-of-home placement [16, 29]. Yet, both studies have methodological limitations, such as the lack of a control group [29] and a retrospective quasi-experimental case-file-analysis design without measures of youth adaptivity [16]. Thus, further research is needed, with more rigorous designs to examine the effects, moderators, and mediators of the InConnection approach.

### Mediators of effectiveness

Treatment mediators identify how treatments work [30]. Three potential mediators are assumed to explain how the collaboration with the YIM in the InConnection approach results in increased youth resilience: social resourcefulness, shared decision making and treatment motivation.

The experience of a supportive relationship with a YIM may increase youth’s social resourcefulness [18], which is the ability to seek help and support from the social network. It is suggested that the positive relationship with a YIM is a safe context for youth to practice and develop their relationship skills, allowing youth to benefit more from the social ties within their networks [18]. Indeed, higher quality mentoring relationships are associated with improved relationships with other adults [31, 32]. Moreover, in a qualitative study [33] youth reported they felt more comfortable seeking help after participation in a mentoring program, suggesting a link between mentoring relationships and social resourcefulness. Social resourcefulness is, in turn, related to positive treatment outcomes, such as increased self-esteem, prosocial behaviors, and reductions in misconduct [31, 32]. As this mediation has only been examined in school-based programs, we will examine whether social resourcefulness mediates the link between YIM and outcomes in the context of care.

Collaboration with a YIM may increase shared-decision making with the client system and broader social network [18]. Shared-decision making means that goalsetting is done in collaboration with the client system and its social network, which is thought to result into personal goals that are set for autonomous reasons [18]. Having personal or self-concordant goals has been associated with goal progress [34], suggesting that shared-decision making may increase treatment effectiveness. The collaboration with a YIM is thought to enhance shared-decision making, as the YIM represents the youth and actively collaborates with the case manager [18], for example in formulating a treatment plan [17]. We thus expect that shared-decision making serves as a mediator of care effectiveness.

The positioning of and collaboration with a YIM is also suggested to contribute to treatment effectiveness through enhanced treatment motivation. It is long known that treatment motivation is an important factor for treatment effectiveness [35]. Self-determination theory [36] suggests that autonomy, competence, and relatedness, which may be present in the context of choosing a YIM, are necessary ingredients of motivation. That is, youth are supported to *autonomously* choose a YIM and participate in shared decision making, as adults such as the social worker believe youth have the *competence* to choose what is right for them. Furthermore, the positioning of a YIM increases the *relatedness* with a supportive figure [37] and others [31, 32]. Mentors also *directly* encourage youth to participate in treatment and achieve challenging treatment goals [21]. Thus, it is expected that youth are more motivated to engage in treatment through the positioning of a YIM.

### Moderators and predictors of effectiveness

In addition to studying the working mechanisms of InConnection, studying moderators and predictors of effectiveness is needed to identify which youth profit most from the approach and under which circumstances [30]. Client characteristics, including age, gender, ethnicity, and socio-economic status, and treatment characteristics, including duration, intensity and content of treatment, will be examined as moderators of effectiveness, because these factors are measured in both treatment groups. As previous research stresses the importance of measuring treatment integrity [38], and suggests that high mentoring relationship quality [24, 25, 39], and the collaborative relationship between the YIM and case manager [18] are associated with positive outcomes, these factors will be examined as predictors.

### Aims and hypotheses

In conclusion, the InConnection approach is a promising treatment program for multi-problem youth, but its effectiveness in comparison to care as usual and potentially important mediators, moderators and predictors have not been investigated yet in a prospective multi-informant study. Similarly, reasons for not positioning a YIM and factors contributing to the successful positioning of a YIM are unknown. Information on the effectiveness, mediators, moderators, predictors, and the positioning of a YIM are assumed to be essential for treatment success.

**Fig. 1:**
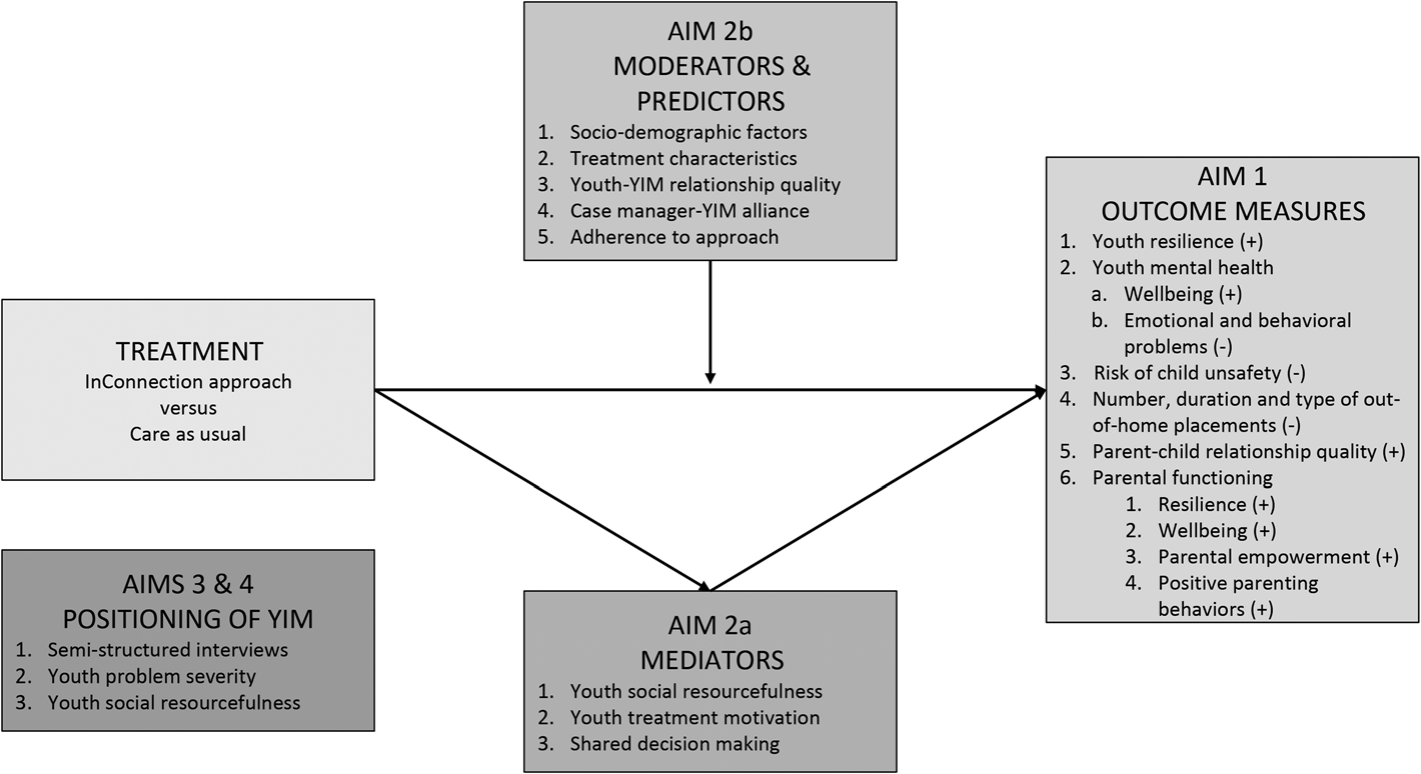
Aims, treatment conditions, outcomes, and potential mediators, moderators and predictors examined in this study

The *Growth in Personal environment* (GRIP) study aims to generate this information. GRIP consists of 1) a prospective, quasi-experimental study to examine the positioning of a YIM, the effectiveness of the InConnection approach, and the mediators, moderators and predictors of effectiveness; and 2) a semi-structured multi-informant interview study to deepen our understanding of why youth do or do not nominate a YIM. Based on prior research and the program theory of YIM [18], hypotheses have been formulated which will be tested with data from both studies. Figure 1 presents this study’s model.

1. The InConnection approach is more effective than care as usual in promoting youth resilience (*primary outcome*), youth mental health, parent-child relationship quality, and parental functioning; and reducing the risk of child unsafety and the number and duration of out-of-home placements (*secondary outcomes*). (Study 1)
2. The effects of the InConnection approach are mediated by social resourcefulness, treatment motivation, and shared decision making; moderated by socio-demographic factors and treatment characteristics; and greater at higher levels of youth-YIM relationship quality, alliance between case manager and YIM, and adherence to the approach. (Study 1)
3. Youth with fewer problems and higher levels of social resourcefulness are more likely to nominate a YIM. (Study 1)
4. Youth who nominate a YIM do so based on the relationship quality and similarities with the YIM. We will exploratively examine why some youth do not nominate a YIM. (Study 2)

## Methods and design

The study is registered at the Netherlands Trial Register (NL7565). The design of the study is according to the guidelines of Helsinki (1964) and its later amendments, and approved by the faculty ethical review board of the Faculty of Social and Behavioral Sciences of Utrecht University (FETC-18-093). The study design is reported in accordance with the SPIRIT 2013 Statement for reporting intervention trials. Participant recruitment started on January 1, 2019 and ends October 1, 2020. The final follow-up measurements are estimated to end in January 2022.

### Design

GRIP is a multi-site study performed at five organizations for youth and family care located in urban areas in the Netherlands. These organizations offer a variety of youth and family care, including – for multi-problem families – the InConnection approach and one or more other approaches for systemic outpatient care (care as usual). Multi-problem families referred to any of these organizations are offered the InConnection approach or care as usual. Allocation to care programs is non-random, as it depends on the availability of care within a specific program (sometimes programs have a waiting list and clients are therefore allocated to the other form of care) and the client’s preference for the content and methods of one care program over the other.

### Study sample

The inclusion criteria are families with: 1) at least one youth aged 10 to 23 years; 2) problems that are considered complex, multiple and severe, and/or previous treatments have not yielded the intended effects, and/or indication for an out-of-home placement; 3) sufficient Dutch proficiency.

#### Quasi-experimental study

For the quasi-experimental study, a total of 300 multi-problem families (*N* = 300) will be included in this study consisting of at least one family member. If approved by the youth, the case manager and the YIM are also approached for participation in the study.

The *N* = 300 included families will consist of *n* = 225 families in the intervention group and *n* = 75 families in the control group. We have chosen for a 3:1 ratio to allow propensity score matching. Participants will be matched on the following characteristics: age, ethnicity, gender, educational level, resilience, and severity of psychopathology, which will result in two comparable groups of *n* = 75 for the analyses.

#### Power analyses

The number of families per group was determined by a priori power analyses using the commonly accepted power level of .80 and α = .05. A power analysis in G*Power 3.1 [40] was performed for the research question on the overall effectiveness of InConnection in terms of primary and secondary outcomes. A total sample size of *n* = 138 is sufficient to identify small effects (*f* = .10) in repeated measures analyses of variance. We estimated the power of the analyses to examine mediation [41] and moderation [42] of intervention effects using R [43]. Our total sample size of *n* = 150 is sufficient to detect mediation in a model with medium correlations (*r* = .30), and to detect moderation in a model with β = .25. Analyses to test prediction of intervention effects and to compare youth who nominated a YIM mentor to youth who did not, will be performed using the intervention group only (*n* = 225). For the regression analyses to test prediction of effectiveness, sample sizes of *n* = 52–65 are required depending on the number of predictors to find a small effect (*f*^2^ = .20) as demonstrated by power analyses in G*Power 3.1 [40]. A sample of *n* = 228 is required for the *t*-test comparing youth who found a YIM to youth who did not, to find a medium effect (*d* = .20). Expectations of effect sizes were based on meta-analyses on the effects of formal [24] and informal [25] mentoring, as well as empirical studies on the effects of YIM in the context of care [27–29].

If the data show a hierarchical structure and require multilevel analyses, we will perform an interim power analysis while recruitment is still active. To estimate power for multilevel analyses, a large number of factors must be estimated, including the means, variances, and covariances for the explanatory variables, the sample sizes at each level and the variances and covariances for the random effects. These values are notoriously difficult to estimate a priori [44]. Therefore, we will use an internal pilot study design to perform an interim power analysis before closing the recruitment phase to determine whether a sufficient sample size has been obtained or whether the recruitment phase should be extended (within the constraints of our project) [45].

#### Interview study

The semi-structured interviews are conducted in a subsample of the intervention group from the quasi-experimental study, whose selection is based on background characteristics, such as age, gender, ethnicity and city, by which we aim to seek the maximum variation in experiences. A total of 10–20 client systems is selected: We select five to ten client systems who nominated a YIM within 6 weeks after the start of the treatment, and five to ten client systems who did not nominate a YIM within this time frame.

### Recruitment

It is estimated by the participating organizations that an average of 22 clients start treatment every month. Given the number of participants to be included in the study (*N* = 300), and taking into account that approximately two thirds of clients give consent for participation, we expect to complete the inclusion period within 21 months (January 2019 to October 2020).

**Fig. 2:**
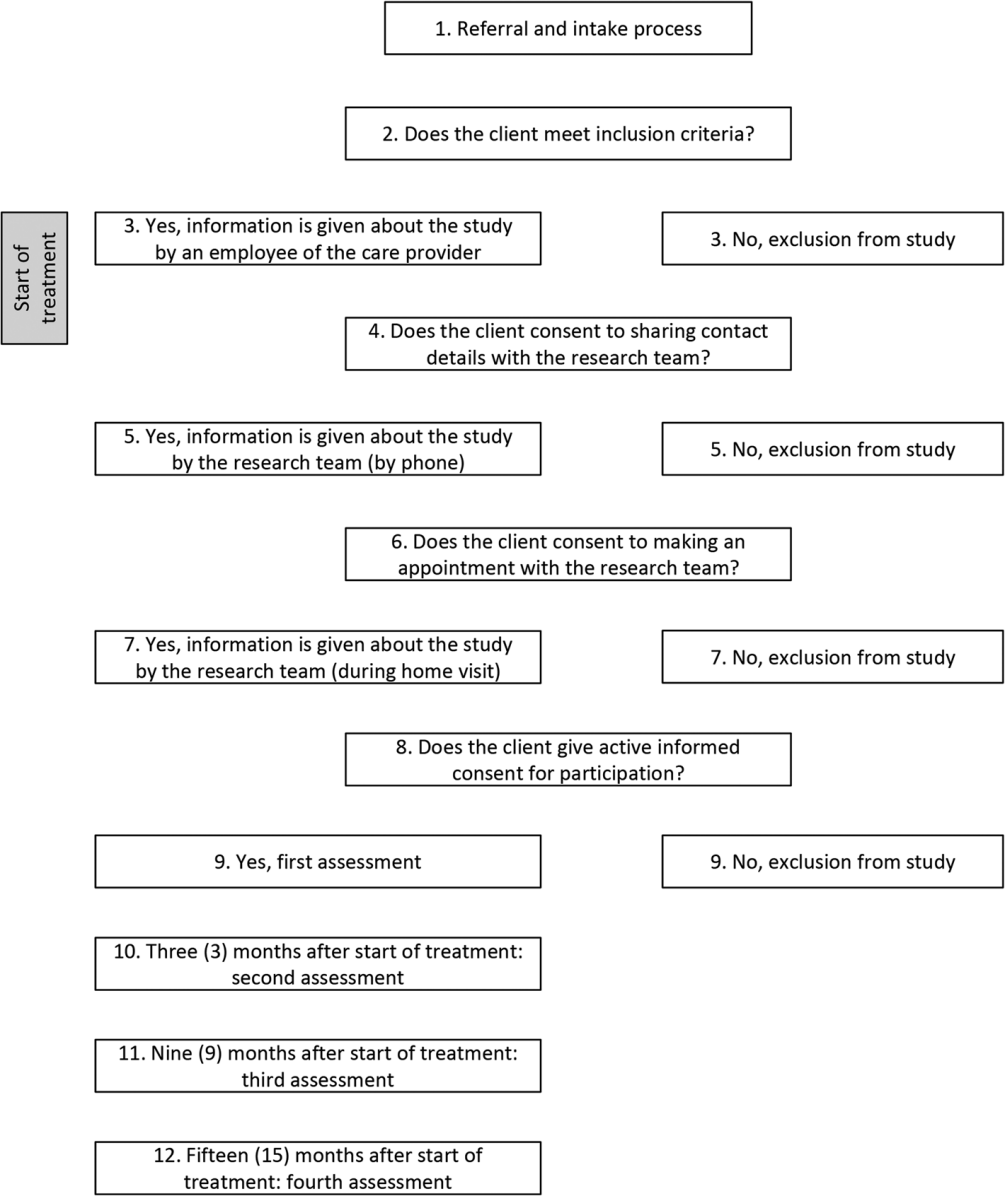
Participants’ flow through the study

Families that start treatment in one of the treatment groups in this study between January 1, 2019 and October 1, 2020 are informed about this study by an employee of the care providing organization, often the case manager. The employee asks verbal permission from the client system to share their contact details with the independent research team. A member of the research team then makes a phone call to the client system, informs the client of the study, and suggests to schedule an appointment with the client, parents and/or YIM to further inform them about the study. Active informed consent for participation is received from youth, parents, and YIMs for their own participation. For youth under the age of 16, active informed consent for their participation is also received from one parent. Participants will receive a financial reward of €50 for completion of the questionnaire assessments and €10 for participation in the interview. See Fig. 2 for the participants’ flow through the study. The frequency of non-response and drop-out will be meticulously recorded for every stage in the study.

### Conditions

#### InConnection approach

The InConnection approach is designed as a systemic outpatient alternative to out-of-home care for youth from multi-problem families. Treatment consists of four phases: 1) *who*, 2) *what*, 3) *how*, and 4) *adaptivity* [17]. The first phase will be discussed in most detail, as this phase is unique to the InConnection approach. In contrast to most other treatment programs, the InConnection team does not start with an analysis of problems. Instead, in the first phase, that is, *who*, the case manager opens the conversation on the value of a YIM and its implications for the family and the professional. The case manager explains that a YIM is someone who is trusted by the youth, someone s/he can go to for support or advice, and/or someone who inspires the youth. The youth is asked to think about who could be this person for him/her. If necessary, the case manager provides more support in identifying a potential YIM, for example by making a social network map. Once youth have identified a potential YIM, this person is nominated by the youth and invited for a meeting with the case manager. The case manager explains what the positioning of a YIM means. If the YIM accepts the position as YIM, all parties meet to discuss issues of confidentiality, privacy, contact frequency, boundaries, and a worst case scenario, which are laid down in a plan of action. The YIM is officially installed when all parties have signed the plan of action [17]. The duration of this phase is on average 1 month.

In the second phase, that is, *what*, all parties give their opinion on what they would like to see changed. The case manager motivates youth, parents, and YIM to discuss the ideal situation. This information is used by the professional to make an analysis of the problem and potential solutions. In the third phase, that is, *how*, all parties work together on formulating a plan of action based on the input from the second phase. The plan of action documents the treatment goals, what support is offered by professionals, such as specialized treatment, and what support is offered by the informal network. In this phase, the plan of action is also executed, and evaluated with all parties every 2 months. The fourth and final phase, *adaptivity*, starts when treatment goals have been met and/or all parties feel that professional support is no longer needed. The case manager poses several questions to the youth, parents, and YIM, such as ‘what changes when professional support ends?’ and ‘what happens to the position of the YIM?’. Once all parties agree on how the family will proceed without professional support, the treatment is concluded [17].

As treatment is tailored to the needs of a family, the treatment varies in duration and content. That is, for youth with more complex needs, the treatment may take 12 months or more, whereas for others the treatment may only take 6 months. To tailor the content to the family’s needs, the treatment teams consist of professionals with different types of expertise: youth and family care, psychiatry, addiction care, and care for people with mild intellectual disabilities. These professionals are trained in delivering the treatment according to the InConnection approach to enhance adherence to the guidelines. The number and combination of treatment techniques used differ across families. A few examples: youth with addiction problems can be offered specialized addiction care; parents who experienced trauma can be offered specialized trauma therapy; and families that experience interpersonal conflicts can be offered systemic counselling [17].

#### Care as usual

Care as usual includes different alternative outpatient treatment programs for multi-problem families. All selected treatment programs are multi-modal systemic family care programs for multi-problem youth and their parents, such as versions of (intensive) family preservation programs. Team members collaborate with other professionals involved in the family (both from within the same organization as from other organizations) to ensure integration of care. Families can thus be enrolled in several treatment programs at the same time. The average duration of the treatment programs is similar to that in the intervention group, that is, approximately six to 12 months. Short-term interventions, such as crisis interventions, are not included.

### Data collection

#### Quantitative data collection

To assess changes in outcomes during treatment, four multi-informant (youth, parent, YIM, and case manager) assessments using questionnaires are conducted: 1) at the start of treatment; 2) after 3 months; 3) after 9 months; and 4) after 15 months. In Table 1, concepts, measures, and informants of all administered instruments are presented. At the first assessment, the youth, parent(s) and YIM complete questionnaires at a chosen location, often at home, in the presence of a member of the research team who assists the participants in answering the questions if problems, such as reading problems, are present. If the participant is 16 years or older and does not experience problems in answering the questions, the subsequent assessments are completed online. To comply with the measures against the coronavirus taken by the Dutch government, we temporarily replaced home visits by phone and video calls. Case managers complete online questionnaires at all assessments. Each assessment takes approximately 30 min to complete. All questionnaires were administered in Dutch.

**Table 1:** Overview of Administered Questionnaires and Informants

Variable	Concept	Measure	Informant			
			Youth	Parent	YIM	Case manager
Primary outcome measure	Youth resilience	CYRM-12	x			
Secondary outcome measures	Wellbeing	WHO-5	x	x		
	Youth emotional and behavioral problems	BPM	x	x	x	
	Out-of-home placements	demographics/ FCU	x	x		
	Parent-child relationship quality	PARA	x	x		
	Parental resilience	ARM-12		x		
	Parental empowerment	FES		x		
	Parenting behaviors	APQ-9		x		
	Risk of child safety	ARIJ				x
Mediators	Social resourcefulness	UCL	x			
	Shared decision making	SRS	x	x		
	Treatment motivation	TMS-F	x			
Moderators	Socio-demographic factors	demographics/ FCU	x	x	x	
	Treatment characteristics	TIFMP				x
Predictors	Youth-YIM relationship quality	PARA, POPS & FIC	x		x	
	Case manager-YIM alliance	WCQ-6			x	
	Adherence to approach	InConnection guidelines				x

*Note*: *CYRM-12* Child and Youth Resilience Measure – Short form, *ARM-12* Adult Resilience Measure – Short form, *PARA* Psychological Availability and Reliance on Adult, *WHO-5* World Health Organization Well-Being Index, *BPM* Brief Problems Monitor, *FES* Family Empowerment Scale, *APQ-9* Alabama Parenting Questionnaire – Short form, *ARIJ* Actuarieel Risicotaxatie Instrument voor Jeugdbescherming [Actuarial Risk Assessment Tool for Protection of Juveniles], *FCU* Family Check-up, *UCL* Utrecht Coping List, *SRS* Session Rating Scale, *TMS-F* Treatment Motivation Scales for Forensic Outpatient Treatment, *POPS* Perceptions of Parents Scale, *FIC* Frequency and Intensity of Contact, *WCQ-6* Work Climate Questionnaire – Short version, and *TIFMP* Taxonomy of Interventions for Families with Multiple Problems

#### Primary outcome measure

The primary outcome measure is resilience of youth as measured by the self-reported Child and Youth Resilience Measure – Short form (CYRM-12), which consists of 12 items [46, 47]. Resilience is the capacity of the individual and its social and physical environment to cope with adversity [15]. The CYRM-12 assesses the resources (individual, relational, communal and cultural) available to individuals that may sustain their resilience. Items are rated on a 5-point scale from 1 = *does not describe me at all* to 5 = *describes me a lot*. Higher scores reflect higher levels of resilience. Internal consistency was satisfactory in the original Canadian sample [46] and a Dutch sample [48] (α = .84 and α = .93, respectively). The CYRM-12 showed sufficient content validity to be used as a cross-cultural screener of resilience [46].

#### Secondary outcome measures

A broad range of secondary outcome measures will be assessed, namely youth and parental wellbeing, youth emotional and behavioral problems, risk of child unsafety, out-of-home placements, parent-child relationship quality, parental resilience, parental empowerment, and parenting behaviors.

Youth and parental wellbeing is measured using the self-reported World Health Organization Well-Being Index (WHO-5), which assesses subjective psychological wellbeing [49]. Youth and parents rate 5 items on a 6-point scale from 0 = *none of the time* to 5 = *all the time*. Higher scores reflect higher levels of wellbeing. The internal consistency and validity were satisfactory in a variety of samples [50], including a Dutch sample (α = .91–.93) [51]. The measure is deemed appropriate for cross-cultural screening purposes and to be used in clinical trials [50].

Youth emotional and behavioral problems are measured using the multi-informant Brief Problems Monitor (BPM). The BPM is the abbreviated version of the Child Behavior Checklist and monitors children’s emotional and behavioral functioning [52]. Youth fill out the self-report version (BPM-Y) and parents and YIMs fill out the parent version (BPM-P). Both versions consist of 19 items, which are rated on a 3-point scale from 0 = *not true* to 2 = *very true*. Higher scores reflect more problems. Psychometric properties of the BPM-Y [53] and BPM-P [52, 53] were adequate in American and Norwegian samples: Internal consistency was high (α = .90 and α = .91, respectively) and validity was satisfactory. Dutch versions of the abbreviated and extended versions of this measure have been developed [54], but the psychometric properties of the BPM have not yet been studied in the Netherlands.

Risk of child unsafety is measured using the Actuarial Risk Assessment Tool for Protection of Juveniles (ARIJ). The ARIJ is a Dutch assessment tool for professionals to assess the future risk of unsafety of children and youth [55]. Case managers rate 32 items on a 3-point scale with 1 = *yes*, 2 = *no*, and ? = *unknown*. (The item “young child, <5 years old” of the original ARIJ has been excluded in this study, as youth participating in our study are 10 years or older.) The risk of future unsafety is scored as low, medium or high. The ARIJ was developed and tested in the Dutch context and has adequate psychometric properties: The items showed adequate interrater and intrarater reliability [56].

The number, duration, and type of previous out-of-home placements experienced by the youth are assessed as part of the demographic questionnaire at the first assessment. Out-of-home placements during the study are assessed at the second, third and fourth assessment, using the same questions. Both youth and parents report on the (history of) out-of-home placements.

Parent-child relationship quality is measured using the Psychological Availability and Reliance on Adult (PARA). The PARA is designed to measure relationship quality in asymmetrical relationships, such as parent-child and mentoring relationships, from an attachment perspective. It measures three aspects of the relationship: availability, reliance, and affective bond [22, 57]. Youth report on the relationship with mothers and fathers separately. Parents individually report on the relationship with their child. Three items of the original affectional bond scale have been deleted, as they were not deemed appropriate for the parent-child relationship (e.g., “You dread knowing you may have another [father/mother] in the future”), resulting in a 16-item scale. Youth and parents report on the 16 items which are identical in content, but phrased from another perspective (i.e., either from the perspective of the child or the parent). Items are rated on a 4-point scale from 1 = *disagree* to 4 = *agree*. Higher scores reflect higher levels of parent-child relationship quality. Its internal consistency (α = .65–.81) and validity were satisfactory for most scales in a Dutch sample [57].

Parental resilience is measured with the self-reported Adult Resilience Measure – Short form (ARM-12) consisting of 12 items [58]. The ARM-12 is an adapted version of the CYRM-12 [46] for use with adults. In contrast to the CYRM-12, psychometric properties of the ARM-12 have not been examined yet.

Parental empowerment is measured using the self-reported Family Empowerment Scale (FES), which measures empowerment in families with children who have emotional, behavioral or mental disorders [59]. In this study, only the Family scale that assesses parents’ perception of empowerment in parenting situations is administered. Parents rate 12 items on a 5-point scale from 1 = *never* to 5 = *always*. Higher scores reflect greater empowerment. Validity of the Family scale was good in American [59, 60] and Dutch [61] samples. The internal consistency has only been examined in an American sample, and was excellent (α = .98) [60].

Parenting behaviors are measured using the self-reported Alabama Parenting Questionnaire – Short form (APQ-9). The APQ-9 measures three main parenting practices in response to child behavioral problems: positive parenting, inconsistent discipline, and poor supervision [62]. Fathers and mothers report on the APQ-9 separately. The APQ-9 consists of 9 items that are rated on a 5-point scale from 1 = *never* to 5 = *always*. Higher scores reflect higher levels of parenting practices in a certain domain. Validity of the APQ-9 was good, but the internal consistency was low (α = .44) in an Australian sample [62]. Yet, a low internal consistency is not necessarily problematic when the purpose is to measure a broad concept using few items, like in the APQ-9. Internal consistency of the extended APQ were low to good in a Dutch sample (α = .48–.80) [63]. The psychometric properties of the APQ-9 have not yet been studied in the Netherlands.

#### Mediators

The following potential mediator variables are assessed: social resourcefulness, shared decision making, and treatment motivation.

Social resourcefulness is assessed using the subscale Seeking Social Support of the Dutch questionnaire Utrecht Coping List (UCL). This subscale measures seeking comfort and understanding from others; to tell someone or ask for help [64]. Youth rate the 6 items on a 4-point scale from 1 = *rarely or never* to 4 = *very often*. Higher scores reflect more social resourcefulness. The internal consistency and validity of the UCL were good in a Dutch sample (α = .70–.82) [64].

Shared decision making is measured using the Session Rating Scale (SRS), which is a brief four-item measure of therapeutic alliance. The items tap into a relational bond between the therapist and client, agreement on the goals of therapy, agreement on the tasks of therapy, and the client’s view of the sessions [65]. The second and third item are used to measure shared decision making. Both youth and parents rate the items on a continuous scale of 10 cm, where the left side indicates a more negative response and the right side indicates a more positive response. Thus, higher scores reflect higher levels of shared decision making. The internal consistency and validity of the SRS including all four items were satisfactory to good in American [65] and Dutch [66] samples (α = .88 and α = .85–.95, respectively).

Treatment motivation of youth is assessed using the self-reported Treatment Motivation Scales for Forensic Outpatient Treatment (TMS-F), which measures the motivation to engage in treatment [67]. Youth rate the 16 items of the subscale Motivation to Engage in Treatment on a 5-point scale from 1 = *strongly disagree* to 5 = *strongly agree*. Higher scores reflect greater treatment motivation. Internal consistency and validity were satisfactory in a Dutch adult sample (α = .88) [67]. Psychometric properties have not yet been studied in youth samples.

#### Moderators

Two categories of potential moderator variables are assessed: socio-demographic factors and treatment characteristics.

Socio-demographic factors are self-reported by youth, parents and YIMs, and include age, gender, educational level, ethnicity, and ethnic identity. Parents and YIMs also report on their income as a measure of socio-economic status.

Treatment characteristics are assessed using the Dutch Taxonomy of Interventions for Families with Multiple Problems (TIFMP), which is developed to register techniques that have been used in the treatment of multi-problem families [68, 69]. The TIFMP includes 53 techniques divided over eight domains: A) assessment and organization of information; B) planning and evaluation; C) working on change; D) teaching parenting skills; E) task support; F) activation of the social network; G) activation of the professional network; and H) maintaining the collaboration. The case manager indicates whether a technique has been used in the period between assessments. If relevant, the case manager indicates to whom the technique was directed (e.g., youth, parent, etc.) and whether a specific intervention has been used (e.g., cognitive behavioral therapy). The TIFMP was developed and tested in the Netherlands, and showed sufficient interrater reliability [69].

#### Predictors

The following potential predictors of effects of the InConnection approach are assessed: YIM-youth relationship quality, case manager-YIM alliance, and InConnection approach treatment integrity. These concepts are only measured in the intervention condition.

YIM-youth relationship quality is assessed using three measures: the PARA [22, 57], an adapted version of the Perceptions of Parents Scale (POPS) [70], and a measure of frequency and intensity of contact. The PARA used to assess the YIM-youth relationship quality is similar to the one used to assess parent-child relationship quality. The only difference is the addition of one of the original items from the affectional bond scale (i.e., “It makes no difference to you who your YIM is”). The POPS measures the perception of the child about its caregiver, including its perception on autonomy support. In this study, the POPS is adjusted to measure the youth’s perception of autonomy support from the YIM. Youth rate 9 items on a 5-point scale from 1 = *strongly disagree* to 5 = *strongly agree*. Higher scores reflect more autonomy supportiveness. The internal consistency was good in an American sample (α = .88–.90) [70]. The first author and a professional translator translated the items from English to Dutch using back translation for the purpose of this study. The third measure taps into yet another aspect of YIM-youth relationship quality: frequency and intensity of contact [39]. This measure was developed for the purpose of this study. YIMs report on the frequency, intensity and types of contact with the youth, parent(s), and case manager.

Case manager-YIM alliance is assessed using the Work Climate Questionnaire – Short version (WCQ-6), which measures the YIMs’ perceptions of the degree of autonomy support from the case managers [71, 72]. YIMs rate six items on a 5-point scale from 1 = *strongly disagree* to 5 = *strongly agree*. Higher scores reflect a better alliance. The extended 15-item WCQ is based on two comparable questionnaires with high internal consistency (α = .92–.96) and good validity in American samples [73, 74]. The first author and a professional translator translated the items from English to Dutch using back translation for the purpose of this study.

To assess the adherence to the InConnection approach, case managers indicate whether they have performed the 21 steps of the InConnection approach in the treatment of multi-problem families [17]. Of these 21 steps, 13 are divided over the four phases of the approach. Two steps should be performed to improve the overall alliance with the family. The final six steps are only performed and reported on if the youth has been placed out of home. The instrument was developed in the Netherlands, and its psychometric properties have not been researched yet.

#### Qualitative data collection

The aim of the semi-structured interviews is to obtain detailed, qualitive information on the YIM nomination process as experienced by the youth, parent(s), YIM and case manager. Of each client system, the youth, parent(s), YIM, and case manager are invited for individual interviews at a location chosen by the participant, which is usually at home or at the care organization. Interviews with client systems who have positioned a YIM take place as soon as possible after positioning. Interviews with client systems who have not positioned a YIM within 6 weeks take place after this time frame has passed. The interviews are conducted by trained researchers and are recorded with the permission of the participant. Interviews are transcribed verbatim by research assistants. The first author, who will conduct the majority of the interviews, developed an interview topic guide based on the nomination process of a YIM in the YIM approach [17] and previous research. Questions about important adults and help-seeking were added based on research on social networks and social support e.g., [75, 76]. Research on formal and informal mentoring helped us to develop questions about potentially important factors on which youth might base their choice for a YIM, such as trust [77], gender [78] and ethnicity [79]. Interview topics and questions are tailored to the experiences of each sample and participant type. Example questions are: “Can you tell me about how you chose [YIM] to be your YIM?” (question for youth with a positioned YIM); “What qualities do you think a successful YIM should have?” (question for youth without a positioned YIM); and “What is the reason that you want to help [youth]?” (question for YIM). For more details on the interview topics for youth, see Appendix A. Interviews last approximately 30 min.

### Data management

Data is collected and stored in accordance to the guidelines of Helsinki (1964) and its later amendments, and guidelines of the faculty ethical review board of the Faculty of Social and Behavioral Sciences of Utrecht University. Collected data is processed and stored anonymously by storing raw data separately from identifiable data.

### Data analyses

#### Quantitative data analyses

Preliminary analyses are conducted using data from the TIFMP to examine differences in intervention techniques between the InConnection approach and care as usual. All statistical analyses will be performed in Mplus [80] using an alpha level of 0.05, following the intention-to-treat principle, but will also be analyzed per protocol. Missing data patterns are checked using Little’s test in SPSS [81]. If missing data are missing completely at random, the default setting in Mplus for handling missing data, that is, full information maximum likelihood, is used.

The data collected have a multilevel structure, as assessments are nested within participants and participants are nested within care organizations. Therefore, we will examine intraclass correlations to test whether there is significant variance at each level. In case of significant variance at multiple levels, multilevel analyses will be performed. In case of no multilevel structure in the data, for example due to low level of variance at the organization level, more parsimonious models without multilevel structure will be performed.

To examine which families in the intervention group are more likely to position a YIM within 6 weeks, a *t*-test is performed, comparing families who positioned a YIM within 6 weeks to those who did not position a YIM. The two groups are compared on severity of problems, social resourcefulness, and background characteristics.

To examine the intervention effects of the InConnection approach vs. care as usual, repeated measures analyses of variance are conducted. Mediators, moderators and predictors of intervention effects will be tested by linear multiple regression analyses. Separate models are conducted for each outcome variable to avoid a decrease in statistical power due to the addition of many variables. We will control for differences in timing of assessments across respondents by using time-variant models and test for the potential influence of covariates, such as socio-demographic factors.

#### Qualitative data analyses

To understand how and why youth selected a YIM, multistep thematic analysis [82] will be conducted in NVivo [83] of interviews with youth, parents, case managers, and YIMs from 10 to 20 client systems. For each client system, interviews will be conducted with the client, parent(s), case manager and (if applicable) a YIM. An initial codebook will be developed by the first author drawing from the interview topic guide and initial impressions of a small number of interview transcripts. The interviews are thematically coded using these initial codes, while the codebook will be continuously evaluated and refined based on themes identified in the coding process. All available interviews within one family are coded together. Once coding for one family is complete, the coder constructs a narrative summary, summarizing and synthesizing the participants’ perspectives and experiences of the YIM selection process. These narrative summaries are then read multiple times to identify themes across families, which are laid down in a conceptually clustered matrix [84].

## Discussion

Giving the lack of convincing evidence for an effective treatment for children of multi-problem families [4, 5], evidence-based approaches are urgently needed. In this article we have presented the protocol of the GRIP study designed to investigate the effectiveness of the InConnection approach, an individualized treatment program for multi-problem families with specific focus on collaboration with the social network. By conducting a prospective quasi-experimental study with propensity score matching, the GRIP study aims to examine the effectiveness of the InConnection approach as well as mediators, moderators and predictors of this effectiveness among multi-problem families. Furthermore, the GRIP study aims to examine the selection process of a YIM in families who have succeeded to find a YIM within 6 weeks and families who have not. With this study, we hope to contribute to the treatment of multi-problem families as well as generate knowledge on mediators, moderators and predictors of treatment effectiveness.

### Strengths and challenges

This study has several strengths. First, the study is conducted under real-life circumstances, thus testing the effectiveness, rather than the efficacy, of the InConnection approach, which optimizes the ecological validity and improves the generalizability into other real-life settings. Furthermore, this study compares two different active treatment conditions that are similar in most aspects, such as the systemic and individualized approach and the intensity and duration of the treatment. This makes it possible to disentangle the effects of the unique components of the InConnection approach, that is, the integrated care offered by a multidisciplinary team and the YIM. Additionally, by also examining mechanisms that can explain these effects (i.e., mediators) and circumstances under which the effects may be weaker or stronger (i.e., moderators), we gain better insight into what works for whom. A second strength is the use of validated measures and a mixed-methods approach, as the GRIP research project consists of a quantitative questionnaire study and a qualitative interview study. This ‘methodological triangulation’ enhances our understanding, helps interpretation and contributes to the strength of the research [85]. A third strength is the use of multiple informants in both the quantitative and qualitative study, as youth, parents, YIMs and case managers are invited to participate. By collecting information from multiple informants the risk of biases is reduced and contextual variations in behaviors, for example between the home and in proximity of the YIM, can be identified [86].

The design of the study also offers potential challenges. First, the inclusion of participants is dependent on the collaboration with the participating organizations. That is, families have to consent to being contacted by the research team, and this consent is to be asked for by the case manager. To ensure that potential participants are requested for consent, the research team has frequent interaction with contact persons within the organizations and monitors the registrations of new clients. A second potential challenge is non-response and drop out due to multiple assessments and informants included in the study. This may be a particular challenge in our hard-to-reach sample of multi-problem families. We have tried to minimize the effort the families have to put into study participation by doing home visits and online assessments. Furthermore, we have an active and experienced research team with a large group of research assistants, who can quickly react to pending non-response in order to increase the response rate. For example, personal reminders are sent if participants have not completed assessments on time. A third potential challenge is that treatment intensity, duration and content may differ between as well as within the two treatment groups. Therefore, the number of face-to-face sessions with the case manager, as well as duration and content of treatment are registered and are taken into account as potential moderating factors. The fourth potential challenge is the fact that participants are not randomly allocated to one of the treatment groups, but rather self-select their preferred treatment program. Therefore, it is possible that differences in effects can be attributed to confounding client characteristics that have not been measured. This may complicate the interpretation of treatment effectiveness. To increase the comparability of the two groups and minimize the potential influence of measured confounding variables, we use propensity score matching.

### Implications for practice

If the InConnection approach is effective in improving resilience and mental health in youth of multi-problem families and parental functioning of their parents, this may increase the developmental chances for youth in these families and improve quality of life for all family members [1]. In addition, since problems in multi-problem families are transmitted across generations [87], effective treatment may break the intergenerational cycle of problems and thereby potentially protect future generations from developing multiple problems. Both directly and indirectly, this would alleviate the financial burden that intensive professional care use by multi-problem families places on society. Thus, successful treatment of multi-problem families benefits family members, their future generations and society.

In addition, the InConnection approach has the potential to be implemented widely and reach many multi-problem families for a number of reasons. First, the approach will be studied in five regions in the Netherlands, possibly demonstrating the flexibility of the approach to be implemented in different areas. Second, the program is designed to be accessible to all multi-problem families with youth in the age of 10–23 years and has no exclusion criteria. Third, although setting up a multidisciplinary team is an investment for care organizations, time (and money) may also be saved by providing direct access to specialized care for families in need.

Finally, this study will contribute to our knowledge on the effects of multidisciplinary care and YIM in this complex target group of multi-problem families, and on factors that mediate, moderate and predict treatment effects. This knowledge could help to improve the care for multi-problem families and care targeted at improving resilience.

## Data Availability

The dataset is accessible by all authors. Access is granted to students or research assistants who assist in data collection for the duration of their research project membership, after signing a confidentiality agreement. The datasets generated and/or analyzed during the current study are not publicly available due to sensitivity and confidentiality of data, but are made available on reasonable request through the EASY online database or a similar service after completion of the study.

## Appendix

**Table 2:** A translated version of the topic list that was used to guide the interview with youth

Section	Topic	Main question	Prompt
Social network scheme	Important adults	To whom can you go for advice?	Can you tell me something about him/her? How important is he/she to you? What kind of advice do you ask him/her?
Attitude	Attitude towards YIM approach	What was your first impression of the YIM approach?	How do you feel about asking for help? Have you asked someone for help before? How do your parents feel about asking someone for help?
The YIM	The YIM	Can you tell me about your YIM?	How did you get know him/her?
		Does he/she help you often?	With what? Can you give an example?
YIM choice process	YIM identification	Who came to mind when you heard about the YIM approach?	Can you tell me more about him/her?
		Can you tell me how you came to the idea to ask him/her to be your YIM?	Who suggested him/her as a YIM? Did everyone agree? Why (not)?
		Did other people come to mind?	Who? Can you tell me more about him/her?
	YIM question	Did you ask him/her to be your YIM?	If yes, how and when did you ask him/her? Were other people present?
		What was his/her response?	How did you feel when he/she said that? Why do you think he/she said yes/no?
	YIM choice	Why did/didn’t you ask him/her to be your YIM?	Why do you think this quality is important for a YIM to possess? Did this quality play a role in the choice for a YIM?
		*If not already mentioned by the participant, questions concerning these topics are raised:*	- Trust - Non-judgmental - Empathy - Dedication - Time and location - Gender - Ethnicity
Changes since having a YIM	Changes in relationship quality	Has your relationship with your YIM changed since he/she has been appointed as YIM?	How?
	Changes in contact frequency	Do you see each other more or less often?	Does your YIM now come to places or meetings to which he/she did not used to go? Where?

## Abbreviations

APQ-9: Alabama Parenting Questionnaire – Short form
ARIJ: Actuarieel Risicotaxatie Instrument voor Jeugdbescherming [Actuarial Risk Assessment Tool for Protection of Juveniles]
ARM-12: Adult Resilience Measure – Short form
BPM: Brief Problems Monitor
CYRM-12: Child and Youth Resilience Measure – Short form
FCU: Family Check-up
FES: Family Empowerment Scale
GRIP: Growth in Personal environment
PARA: Psychological Availability and Reliance on Adult
POPS: Perceptions of Parents Scale
SRS: Session Rating Scale
TIFMP: Taxonomy of Interventions for Families with Multiple Problems
TMS-F: Treatment Motivation Scales for Forensic Outpatient Treatment
UCL: Utrecht Coping List
WCQ-6: Work Climate Questionnaire – Short version
WHO-5: World Health Organization Well-Being Index
YIM: Youth Initiated Mentor *or* Youth Initiated Mentoring
